# Relevance of the College of American Pathologists guideline for validating whole slide imaging for diagnostic purposes to cytopathology

**DOI:** 10.1111/cyt.13178

**Published:** 2022-09-26

**Authors:** Pietro Antonini, Nicola Santonicco, Liron Pantanowitz, Ilaria Girolami, Paola Chiara Rizzo, Matteo Brunelli, Claudio Bellevicine, Elena Vigliar, Giovanni Negri, Giancarlo Troncone, Guido Fadda, Anil Parwani, Stefano Marletta, Albino Eccher

**Affiliations:** ^1^ Section of Pathology, Department of Diagnostics and Public Health University and Hospital Trust of Verona Verona Italy; ^2^ Department of Pathology University of Michigan Ann Arbor Michigan USA; ^3^ Department of Pathology Provincial Hospital of Bolzano (SABES‐ASDAA) Bolzano‐Bozen Italy; ^4^ Public Health University of Naples Federico II Naples Italy; ^5^ Section of Pathological Anatomy, Department of Human Pathology in Adulthood and Childhood "G. Barresi”, University Hospital G. Martino University of Messina Messina Italy; ^6^ Department of Pathology The Ohio State University Columbus Ohio USA; ^7^ Department of Pathology and Diagnostics University and Hospital Trust of Verona Verona Italy

**Keywords:** CAP guideline, cytopathology, digital pathology, systematic review, validation, WSI

## Abstract

Whole slide imaging (WSI) allows pathologists to view virtual versions of slides on computer monitors. With increasing adoption of digital pathology, laboratories have begun to validate their WSI systems for diagnostic purposes according to reference guidelines. Among these the College of American Pathologists (CAP) guideline includes three strong recommendations (SRs) and nine good practice statements (GPSs). To date, the application of WSI to cytopathology has been beyond the scope of the CAP guideline due to limited evidence. Herein we systematically reviewed the published literature on WSI validation studies in cytology. A systematic search was carried out in PubMed‐MEDLINE and Embase databases up to November 2021 to identify all publications regarding validation of WSI in cytology. Each article was reviewed to determine if SRs and/or GPSs recommended by the CAP guideline were adequately satisfied. Of 3963 retrieved articles, 25 were included. Only 4/25 studies (16%) satisfied all three SRs, with only one publication (1/25, 4%) fulfilling all three SRs and nine GPSs. Lack of a suitable validation dataset was the main missing SR (16/25, 64%) and less than a third of the studies reported intra‐observer variability data (7/25, 28%). Whilst the CAP guideline for WSI validation in clinical practice helped the widespread adoption of digital pathology, more evidence is required to routinely employ WSI for diagnostic purposes in cytopathology practice. More dedicated validation studies satisfying all SRs and/or GPSs recommended by the CAP are needed to help expedite the use of WSI for primary diagnosis in cytopathology.

## Graphical abstract

To date, the application of WSI to cytopathology has been beyond the scope of the CAP guideline, due to limited evidence. Whilst the CAP guideline for WSI validation in clinical practice helped the widespread adoption of digital pathology, more evidence is required to routinely employ WSI for diagnostic purposes in cytopathology practice.

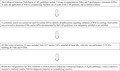

## 1 INTRODUCTION

Digital pathology consists of viewing, sharing, and/or analysing digitised pathology glass slides employing computer‐based technology.^1^ There are numerous clinical (e.g., primary diagnosis, telepathology, image analysis) and non‐clinical (e.g., research, education) applications of digital pathology. Imaging technology related to digital pathology has evolved over time, from static images (microphotographs of a field of view on a slide) to dynamic images (transmission of images in real time), and more recently to whole slide imaging (WSI). WSI technology refers to scanning glass slides to generate digital slides that can be viewed on a computer monitor to recreate a virtual experience that is similar to examining the glass slides with a traditional light microscope.^2^

To demonstrate that this technology works safely for diagnostic patient care and that it can accordingly be adopted for routine clinical work, WSI systems should ideally undergo validation before deployment in clinical service. The crux of such a validation study is to ensure that pathologists' diagnoses using WSI are as accurate as those rendered with glass slides and a light microscope. To assist pathology laboratories with this validation process, in 2013 the College of American Pathologists (CAP) published a specific guideline on how to validate WSI for diagnostic purposes. The CAP guideline incorporated 12 statements to guide pathology laboratories.^3^ The CAP guideline was subsequently updated in 2021,^4^ and differed from the previous publication because a Grading of Recommendation Assessment, Development, and Evaluation (GRADE)^5^ framework was adopted to evaluate available evidence. Moreover, the concept of good practice statements (GPS) was introduced. GPSs differ from strong recommendations (SRs) because while they support important issues they lack the published evidence typically needed for a recommendation. The updated CAP guideline comprising three SRs and nine GPSs is summarised in Table 1.

**TABLE 1:** Strong recommendations (SRs) and good practice statements (GPSs) from the 2021 College of American Pathologists guideline for validation of whole slide imaging systems

Item	Description
SR 1	The validation process should include a sample set of at least 60 cases for one application, or use case (e.g., haematoxylin–eosin–stained sections of fixed tissue, frozen sections, haematology), that reflect the spectrum and complexity of specimen types and diagnoses likely to be encountered during routine practice. The validation should include another 20 cases to cover additional applications such as immunohistochemistry or other special stains if these applications are relevant to an intended use and were not included in the 60 cases mentioned above.
SR 2	The validation study should establish diagnostic concordance between digital and glass slides for the same observer (i.e., intra‐observer variability). If concordance is less than 95%, laboratories should investigate and attempt to remedy the cause.
SR 3	A washout period of at least 2 weeks should occur between viewing digital and glass slides
GPS 1	All pathology laboratories implementing WSI technology for clinical diagnostic purposes should carry out their own validation studies.
GPS 2	Validation should be appropriate for and applicable to the intended clinical use and clinical setting of the application in which WSI will be used. Validation of WSI systems should involve specimen preparation types relevant to intended use (e.g., formalin‐fixed, paraffin‐embedded tissue; frozen tissue; immunohistochemical stains). If a new application for WSI is contemplated, and it differs materially from the previously validated use, a separate validation for the new application should be performed.
GPS 3	The validation study should closely emulate the real‐world clinical environment in which the technology will be used.
GPS 4	The validation study should encompass the entire WSI system. It is not necessary to separately validate each individual component (eg, computer hardware, monitor, network, scanner) of the system or the individual steps of the digital imaging process.
GPS 5	Laboratories should have procedures in place to address changes to the WSI system that could impact clinical results.
GPS 6	Pathologists adequately trained to use the WSI system must be involved in the validation process.
GPS 7	The validation process should confirm all of the material present on a glass slide to be scanned is included in the digital image.
GPS 8	Documentation should be maintained recording the method, measurements, and final approval of validation for the WSI system to be used in the anatomic pathology laboratory.
GPS 9	Pathologists should review cases/slides in a validation set in random order. This applies to both the review modality (ie, glass slides or digital) and the order in which slides/cases are reviewed within each modality.

Most validation guidelines related to WSI for diagnostic use, including the aforementioned published CAP recommendations, do not specifically include cytology. In fact, the authors of the CAP guideline underline that at the time of publication, due to lack of published evidence, validation of WSI in cytology was considered beyond the scope. Indeed, the adoption of digital cytology has lagged behind that of digital histopathology for several reasons, such as the difficulty of scanning cytology material on glass slides in different focal planes using Z‐stacking.^6^ Not surprisingly, published clinical validation studies in cytology are less numerous than those involving surgical pathology.

The aim of this study was accordingly to investigate the published literature concerning the validation of WSI systems specifically in cytology, with reference to the CAP guideline.

## 2 MATERIALS AND METHODS

### 2.1 Literature search and article screening

The review question was formulated according to a Population, Index, Comparator, Outcome (PICO) model. Population was represented by a series of cytology cases collected retrospectively or prospectively for the validation study; the Index was the WSI modality for pathology cases, while the Comparator was represented by conventional light microscopy. Outcome was represented by concordance between a diagnosis rendered with WSI and light microscopy, the latter being taken as the reference standard. The main aim of the study was to investigate the adherence of validation studies for WSI in cytology to the CAP guideline. Studies represented by abstract only with limited information were excluded.

A systematic review was conducted according to standard methods and reporting in accordance with the Preferred Reporting Items for Systematic reviews and Meta‐Analysis (PRISMA).^7^ The databases PubMed and Embase were systematically searched up to 20 November 2021 to identify any article regarding a validation study of WSI in cytology. The search strategy comprised combinations of the terms “digital pathology,” “validation,” and “cytology” with their conceptual aliases and variations, adequately adapted to the two databases' search engines. Four authors (AE, IG, NS, PA) independently reviewed all article titles and abstracts with the aid of the Rayyan reference manager web application.^8^

Papers dealing with digital pathology other than human cytology (e.g., histopathology, frozen sections of surgical specimens, etc.), with static and dynamic images, or with animal or experimental models, were excluded, as well as papers in languages other than English. Full texts of the articles fulfilling initial screening criteria were acquired and reviewed against the eligibility criteria. Any disagreement with respect to inclusion of a particular article was resolved by consensus.

### 2.2 Data extraction

Two investigators (SN, PA) independently extracted data from the included studies with a standardised form. Data extracted included: author(s) and publication year, country of origin for the research, total number of cytological cases, site(s) of origin of the cytological material, and compliance with the CAP guideline criteria for SRs and GPSs.

## 3 RESULTS

### 3.1 Overview of the papers

**FIGURE 1:**
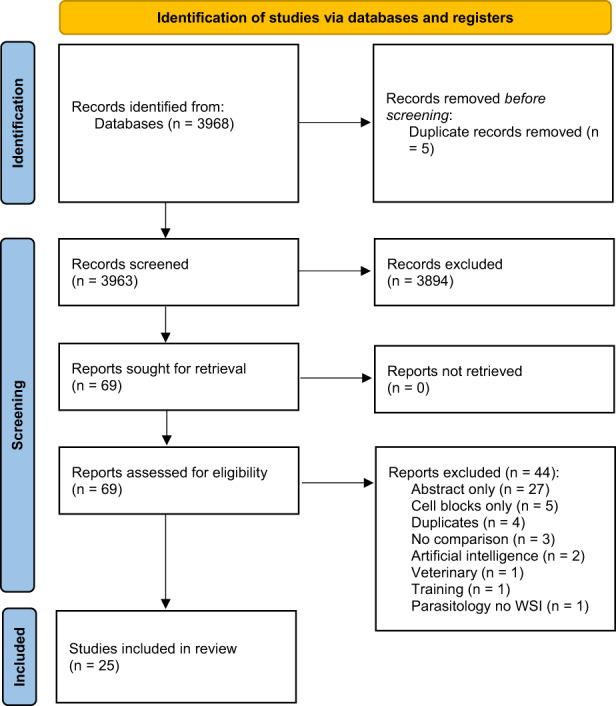
Search flow diagram, adapted from the PRISMA flow diagram template (Page et al^7^). PRISMA, Preferred Reporting Items for Systematic reviews and Meta‐Analysis

A flow diagram of the screening, selection, and exclusion of articles for this review is shown in Figure 1. Briefly, 3963 papers were found and screened with the aid of the Rayyan reference manager web application.^8^ After title and abstract screening were undertaken, 69 papers were selected as potentially relevant to the review and after subsequent full text assessment 44 articles were then excluded. Thus, overall 25 papers were included in our review, representing studies published between 2001 and 2021. A cumulative total of 1994 cytological cases were included (ranging from 5 to 505 cases per study), and comprised case series from Australia,^9^ Canada,^10^ China,^11^ Colombia,^12^ India,^13^ Italy,^14^ Japan,^15^ the Netherlands,^16^ Norway,^17^, ^18^ Poland,^19^ Portugal,^20^, ^21^ Taiwan,^22^ the UK,^23^ and the USA.^23^, ^24^, ^25^, ^26^, ^27^, ^28^, ^29^, ^30^, ^31^, ^32^. Of the 25 papers included, eight (32%) dealt with gynaecological cytology,^14^, ^16^, ^23^, ^24^, ^25^, ^27^, ^32^, ^33^ and the remainder (68%) with non‐gynaecological cytology including three with thyroid cytology,^20^, ^21^, ^30^ one with thoracic cytology,^19^ one with central nervous system cytology,^10^ one with breast cytology,^15^ one with peripheral blood smears,^28^ and ten with specimens derived from different anatomic sites.^9^, ^11^, ^12^, ^13^, ^17^, ^18^, ^22^, ^26^, ^29^, ^30^, ^31^

### 3.2 Assessment of strong recommendations (SRs) and good practice statements (GPSs)

Only 4/25 papers (16%) satisfied the criteria for all three SRs,^13^, ^17^, ^28^, ^32^ and only 1/25 paper (4%) satisfied all three SRs and nine GPSs.^13^ Moreover, 10/25 papers (40%) did not indicate compliance for even a single SR, while 24/25 (96%) failed to demonstrate compliance for at least one GPS. 9/25 (36%) papers addressed a single SR only, while 16/25(64%) did not address any SR, making SRs the most ignored parameters. On the other hand, 25/25 studies (100%) satisfied GPS 1, 2, and 4. An overall depiction of the included studies and their compliance with specific SRs and GPSs is provided in Table 2.

**TABLE 2:** Characteristics of the included studies and their compliance with each SR and GPS from the College of American Pathologists guideline for validation of whole slide imaging systems

Authors	Year	Country	Case number	Anatomic site	Stain	Digital system	SR 1	SR 2	SR 3	GPS 1	GPS 2	GPS 3	GPS 4	GPS 5	GPS 6	GPS 7	GPS 8	GPS 9
Steinberg^24^	2001	USA	10	Cervicovaginal	Papanicolaou	BLISS™ system; WebSlide Browser™												
Marchewsky^23^	2006	USA	20	Cervicovaginal	Papanicolaou	ScanScope (Aperio); VG700b displays												
Dee^25^	2007	USA	5	Cervicovaginal	Not specified	MicroBrightField software												
Slodkowska^19^	2009	Poland	28	Thoracic	Not specified	Coolscope (Nikon); ScanScope (Aperio)												
Evered^33^	2010	UK	20	Cervicovaginal	Not specified	NanoZoomer HT (Hamamatsu Photonic KK)												
Gould^10^	2012	Canada	30	Brain	Not specified	NanoZoomer HT 2.0 (Hamamatsu Photonic KK); NDP.view software; HP L2245 wg monitor												
House^26^	2013	USA	21	Mixed	Papanicolaou, Diff‐Quik, H&E, Gomori Methenamine Silver	ScanScope XT (Aperio); Spectrum and ImageScope software (Aperio)												
Wright^27^	2013	USA	11	Cervicovaginal	Papanicolaou	BioImagene iScan Coreo Au 3.0 (Ventana)												
Donnelly^32^	2013	USA	192	Cervicovaginal	Not specified	BioImagene iScan Coreo Au (Ventana); ImageViewer 3.0.0.0 software (Ventana)												
Gerhard^20^	2013	Portugal	222	Thyroid	Diff Quik	NanoZoomer HT 2.0 (Hamamatsu)												
Gomez‐Gelvezet^28^	2015	USA	100	Peripheral blood smears	Wright‐Giemsa	ScanScope (Aperio); ImageScope software (Aperio)												
Vodovnik^18^	2015	Norway	36	Mixed	Romanowsky or Romanowsky/Papanicolaou	ScanScope AT turbo (Leica); ImageScope software (Leica); SymPathy LIMS (Tieto)												
Hang^22^	2015	Taiwan	10	Mixed	Papanicolaou	SCN400 (Leica); Aperio eSlide Manager (Leica)												
Mukherjee^30^	2015	USA	12	Thyroid	Papanicolaou	iScan Coreo Au (Ventana); ImageViewer 3.1 software (Ventana)												
Arnold^31^	2015	USA	21	Paediatric	Not specified	Model XT (Aperio); ImageScope software (Leica)												
Hanna^29^	2017	USA	30	Mixed	Papanicolaou, Diff‐Quik, H&E	ScanScope XT (Leica); ImageScope software (Leica)												
Vodovnik^17^	2018	Norway	204	Mixed	Romanowsky	ScanScope AT Turbo (Aperio); ImageScope software (Aperio); SymPathy LS (Tieto)												
Bongaerts^16^	2018	Netherlands	505	Cervix	Not specified	250 Flash II scanner (3D Histech); Pannoramic viewer software (3D Histech)												
Huang^11^	2018	China	100	Mixed	Not specified	200 Android smartphones; Aperio AT2												
Ross^9^	2018	Australia	56	Mixed	Papanicolaou, Romanowsky	Aperio ScanScope XT (Leica); ImageScope software (Aperio); Collibio web links (Pixcelldata)												
Yamashiro^15^	2019	Japan	23	Breast	Not specified	Panoptiq software												
Mosquera‐Zamudio^12^	2019	Colombia	17	Mixed	Papanicolaou	NanoZoomer HT 2.0 (Hamamatsu Photonics); Panoptiq system												
Canberk^21^	2020	Portugal	227	Thyroid	Papanicolaou, Diff‐Quik	P250 Flash III; PMidi; PDesk (3DHistech)												
Rajaganesanet^13^	2021	India	60	Mixed	Papanicolaou, May–Grunwald–Giemsa	Not specified												
Negri^14^	2021	Italy	34	Cervix	Papanicolaou	D‐Sight scanner (Menarini); JVSview software												

*Note*: Shadings indicate whether the study satisfied (green), did not satisfy (red), or did not report data (yellow) for each SR or GPS. Abbreviations: SR, strong recommendation; GPS, good practice statement.

## 4 DISCUSSION

Whole slide imaging technology involves the acquisition of digital images of entire pathology glass slides.^34^ WSI has numerous benefits such as portability of pathologists, easy sharing of digital slides, side‐by‐side comparison of slides on a monitor, image analysis, and several other useful applications.^35^ As a result, WSI has gained popularity for clinical purposes such as teleconsultation, as well as educational purposes and research activity.^36^ Systematic reviews on the concordance of WSI versus viewing glass slides using light microscopy have demonstrated that the overall diagnostic concordance between these two modalities is greater than 90%, sometimes with an excellent κ coefficient.^37^, ^38^ However, the application of WSI for cytology has been problematic due to several technical reasons (e.g., cytology smears may cover the entire glass slide surface, cytology material has areas of variable thickness, there may be obscuring material, and cell clusters in three‐dimensions make it difficult to focus in just one plane).^39^ For these reasons, publications regarding WSI in cytology are limited and concordance results with glass slides reported for digital cytology versus histology differ.^39^ Nevertheless, this gap is closing as more publications provide data supporting the diagnostic use of WSI for cytopathology.^40^, ^41^

### 4.1 Strong recommendations (SRs)

SR 1 states that the validation process should include a sample set of at least 60 cases for one application or use case that reflect the spectrum and complexity of specimen types and diagnoses likely to be encountered during routine practice. The validation process should include another 20 cases to cover additional applications such as immunohistochemistry or other special stains if these applications are relevant. However, the Royal College of Pathologists best practice recommendations for implementing digital pathology in 2018 stated that the sample size and duration of the validation process can vary according to specific circumstances.^42^ This is true particularly for studies that focus on rare pathologies for which it may prove difficult to recruit enough cases to meet SR 1.

Our systematic review indicates that 68% of included studies did not meet the required number of 60 cases, with 24% of the studies reporting less than 20 cases, and with a minimum of only five cases in one study.^25^ In our opinion, sample size is an important criterion and we accordingly recommend this be adhered to in future validation studies of WSI in cytology. As explained by the CAP in their updated guideline, a reasonable number of cases will be needed in the validation process in order to include enough cases that represent the entire spectrum and proportion of diagnoses likely to be encountered in a particular clinical setting.

SR 2 states that validation studies should establish diagnostic concordance between digital and glass slides for the same observer. The original diagnoses for selected cases to be used may have been made by pathologists other than those completing the validation, thereby providing additional usable information on inter‐observer variability. The recommendation from the CAP is that, although all discordances between WSI and glass slide diagnoses discovered during the validation need to be reconciled, laboratories should only be concerned if their overall WSI‐to‐glass slide concordance is less than 95%. More than half (16/25, 64%) of the studies in our review indicated intra‐observer concordance below this standard. This may be partially explained by the fact that most of the papers were conducted in a setting where the diagnosis with light microscopy and diagnosis with digital slides were rendered in different places by different pathologists. In 2015 Vodovnik et al^18^ compared the timing of digital and microscopic diagnosis in routine practice, finding that digital cases were diagnosed more quickly. However, no quantitative data about concordance between digital and light microscopy was assessed.

Intra‐observer variability was only established in 28% of the papers. Donnelly et al^32^ evaluated 192 gynaecological cases, and reported an overall intra‐rater concordance for each of the five investigators in their study, ranging from 89% to 97%, with only one meeting the 95% criterion recommended by the CAP. Moreover, in the work by Gerhard et al^20^ intra‐observer variability was only reported for one of the two physicians, with an intra‐observer concordance of 77.5% involving 222 thyroid cases. Gomez‐Gelvez et al^28^ assessed 100 peripheral blood smears and reported an intra‐observer variability for each of four participants, with concordance rates ranging from 88% to 94%. Discordances reported that did not impact patient management (defined as minor discordances) were 8%, 8%, 4%, and 4% for the separate evaluators; conversely, major discordances potentially affecting patient management were 4%, 2%, 2%, and 4%, respectively, for each reader.

A more accurate intra‐observer concordance evaluation in terms of Cohen's Kappa coefficient was reported in the validation study by Rajaganesan et al^13^ that included 60 cytology cases, with two pathologists who reported almost perfect concordance (k = 0.8), another two pathologists who had substantial intra‐observer agreement (k = 0.6‐0.8), and another one that had moderate concordance (k = 0.4‐0.6). Diagnostic concordance in the study by Hanna et al^29^ was assessed on 30 cases, including five cell blocks, and compared the results for two digital systems (Panoptiq and an Aperio system). Indeed, as recommend by the CAP, each scanner requires its own set of validation cases. Furthermore, the study by Vodovnik et al^17^ that included 600 total cases, 204 of which were cytology specimens, did not report on concordance rates. Only six of their cases revealed minor discordances, none of which involved the cytopathology cases. Hence, supposedly the intra‐observer concordance for their cytology cases was 100%. Mukherjee et al^30^ reported an intra‐observer concordance for 12 thyroid cases that were scanned with three, five, and seven focal planes. Their intra‐observer concordance ranged from 92% to 100%.

Finally, SR 3 states that a washout period of at least 2 weeks should occur between viewing digital and glass slides. This recommendation is intended to address the issue of recall bias when cases are reviewed using different modalities by the same observer.^43^ A significant proportion (44%) of the studies included in our review met this criterion, as all studies reporting intra‐observer variability respected at least a two‐week washout interval, apart from the publication by Mukherjee et al^30^ where the washout period was 2 days.

### 4.2 Good practice statements (GPSs)

In the CAP guideline, GRADE introduced the concept of GPS for several issues where published evidence was lacking to support specific recommendations. Overall, 50% of all the GPSs were met, compliance was not specified for 47% of GPSs, and 3% were not satisfied at all. Evaluation of included publications for fulfilment of GPSs was difficult given that extensive descriptions of the study setting were not always available. GPS 1, 2, and 4 were satisfied in all 25 studies. GPS 3 states that the validation study should closely emulate the real‐world clinical environment in which the technology will be used and laboratories are free to incorporate whatever they feel would be appropriate to achieve this goal. In our review, while six studies failed to report, only two studies provided data that did not comply with this parameter. Namely, Bongaerts et al,^16^ while evaluating WSI for cervical cytology, enriched their samples with high‐grade squamous intraepithelial lesion (HSIL) cases, thereby increasing the vigilance to identify such lesions. Also, Dee et al^25^ evaluated the effectiveness of 3‐D versus 2‐D virtual microscopy as adjuncts to education and assessment in cervical cytology. Although 3‐D virtual microscopy systems were on the market or under development at the time of the study, they were not yet were fully integrated for a rapid pan and view with instantaneous focusing capability. Results reported a general consensus that virtual cervical cytology slides would be a useful augmentation to education and testing; however, there was minimal enthusiasm for using virtual slides to replace glass slides. GPS 5 states that laboratories should have procedures in place to address changes to the WSI system that could impact clinical results. Only two studies fulfilled this criterion.

GPS 6 states that pathologists adequately trained to use a WSI system must be involved in the validation process. As clearly reported by the CAP, this was not an evidence‐based recommendation. Moreover, no metrics were suggested to determine technical competency of pathologists using WSI systems. Instead, adequate training is best defined at the discretion of the laboratory medical director.^4^ The same applies for the number of pathologists participating in the validation process. In our review, for 64% of the studies pathologist training and competency was not specified. Of interest, Hang et al^22^ found that participants from educational programs could make diagnostic interpretations using WSI even without prior experience. Similarly, Rajaganesan et al^13^ showed that pathologists can adapt to new technologies irrespective of the system used. Similarly, House et al^26^ had cytotechnologists with and without digital experience participate in their validation study. On the other hand, in the work by Dee et al,^25^ 28 out of 79 evaluators were students, without any routine diagnostic experience.

GPS 7 states that the validation process should confirm all the material present on a glass slide is eventually included in the digital image. The CAP guideline highlights the possibility for scans to be missing some or all of the material present on a glass slide, which may have serious clinical and legal consequences. Possible solutions for this issue include digitising all tissue blocks for comparison, viewing thumbnails of entire scanned slides prior to sign out, introducing a quality control step for a technician to check all scanned slides to verify that all material was completely scanned, or using image analysis software to detect missing tissue on virtual slides. In our review, 7/25 studies (28%) met this criterion, 3/25 (12%) did not, and 15/25 (60%) did not specify their compliance. For example, Steinberg et al^24^ specified that only 20%‐30% of the cellular area of each slide was digitised. Gomez‐Gelvez et al^28^ specified that they did not fulfil GPS 7 due to impractically large‐size files which were too hard to search for scan failures. Similarly, Mukherjee et al^30^ scanned less than 40% of each slide, especially if the smear covered more than 75% of the slide surface, to reduce scan time and file sizes. Wright et al^27^ reported that in their study the entire area occupied in SurePath slides was scanned while the edges of ThinPrep slides were not scanned.

GPS 8 states that documentation should be maintained recording the method, measurements, and final approval of validation for the WSI system to be used in the anatomic pathology laboratory. Most of the studies, 24/25 (96%), did not specify their compliance with this recommendation. Lastly, GPS 9 states that pathologists should review cases/slides in random order for validation purposes. With regard to our review, only 3/25 studies (12%) met this criterion. However, it should be mentioned that the CAP publication states there is no specific evidence that changing the order in which cases/slides are reviewed actually influences the data collected, so that the relative weight of this GPS on the validation process is in question.

### 4.3 Final considerations and limitations of the study

This review aimed to highlight the strengths and limitations of various validation studies specific to WSI diagnostic use in cytopathology according to the SRs and GPSs of the CAP guideline. The strength of our systematic review was the inclusion of validation studies conducted according to the CAP guideline. For the SRs, more than half of the included studies did not meet recommendations concerning at least 60 cases to be utilised, reporting adequate concordance measures, and using the recommended two‐week washout period to read cases on different modalities. For the GPSs, adherence in included studies was variable. Of note, the CAP guideline is only a recommendation and thus is not mandatory for all cytology laboratories to follow. A limitation of our review is the small number of studies included. Also, the reason for missing data was not apparent in all of the articles reviewed. Another limitation was the difficulty we had with homogenously evaluating SRs and GPSs. SRs are numerical parameters, which can be evaluated objectively (i.e., number of cases, washout period of at least 2 weeks), whereas GPSs are more subjective items which may accordingly be interpreted differently by reviewers.

## 5 CONCLUSION

Increasing global experience and published data support the diagnostic use of WSI for cytopathology. However, extensive validation studies for such diagnostic use in routine cytology practice is still required. Most publications to date about validation studies using WSI for diagnostic use in cytology failed to satisfy many of the recommendations established in the CAP guideline. We accordingly recommend that future validation studies in this field be conducted with more a rigorous study design, in terms of better adherence to the guideline, which will help generate robust evidence to support the successful deployment of WSI for diagnostic use in cytology. Finally, WSI is the first step towards the implementing of artificial intelligence‐aided diagnostics, which may be particularly useful in screening cytology, where most of the cases are negative. This will create the need for new validation criteria which will have to be included in future recommendations and guidelines.

## AUTHOR CONTRIBUTIONS

All authors participated in the conception and design or analysis and interpretation of the data. All authors contributed to the drafting of the manuscript and approved the final version of the manuscript.

## CONFLICT OF INTEREST

The authors declare no conflicts of interest.

## Data Availability

Data sharing is not applicable to this article as no new data were created or analysed in this study.
